# Deprivation-specific life tables using multivariable flexible modelling – trends from 2000–2002 to 2010–2012, Portugal

**DOI:** 10.1186/s12889-019-6579-6

**Published:** 2019-03-07

**Authors:** Luís Antunes, Denisa Mendonça, Ana Isabel Ribeiro, Camille Maringe, Bernard Rachet

**Affiliations:** 1 Grupo de Epidemiologia do Cancro, Centro de Investigação do IPO Porto (CI-IPOP), Instituto Português de Oncologia do Porto (IPO Porto), Rua Dr. António Bernardino de Almeida, 4200-072 Porto, Portugal; 2 Faculdade de Ciências, Universidade do Porto, Rua do Campo Alegre 1021/1055, 4169-007 Porto, Portugal; 3 EPIUnit - Instituto de Saúde Pública, Universidade do Porto, Rua das Taipas, n° 135, 4050-600 Porto, Portugal; 4 Instituto de Ciências Biomédicas Abel Salazar, Universidade do Porto, Rua Jorge de Viterbo Ferreira 228, 4050-313 Porto, Portugal; 5 Departamento de Ciências da Saúde Pública e Forenses e Educação Médica, Faculdade de Medicina, Universidade do Porto, Porto, Portugal; 6 Cancer Survival Group, Department of Non-Communicable Disease Epidemiology, Faculty of Epidemiology and Population Health, London School of Hygiene and Tropical Medicine, London, UK

**Keywords:** Life-tables, Deprivation, Multivariable modelling, Socioeconomic factors, Health inequalities

## Abstract

**Background:**

Completing mortality data by information on possible socioeconomic inequalities in mortality is crucial for policy planning. The aim of this study was to build deprivation-specific life tables using the Portuguese version of the European Deprivation Index (EDI) as a measure of area-level socioeconomic deprivation, and to evaluate mortality trends between the periods 2000–2002 and 2010–2012.

**Methods:**

Statistics Portugal provided the counts of deaths and population by sex, age group, calendar year and area of residence (parish). A socioeconomic deprivation level was assigned to each parish according to the quintile of their national EDI distribution. Death counts were modelled within the generalised linear model framework as a function of age, deprivation level and calendar period. Mortality Rate Ratios (MRR) were estimated to evaluate variations in mortality between deprivation groups and periods.

**Results:**

Life expectancy at birth increased from 74.0 and 80.9 years in 2000–2002, for men and women, respectively, and to 77.6 and 83.8 years in 2010–2012. Yet, life expectancy at birth differed by deprivation, with, compared to least deprived population, a deficit of about 2 (men) and 1 (women) years among most deprived in the whole study period. The higher mortality experienced by most deprived groups at birth (in 2010–2012, mortality rate ratios of 1.74 and 1.29 in men and women, respectively) progressively disappeared with increasing age.

**Conclusions:**

Persistent differences in mortality and life expectancy were observed according to ecological socioeconomic deprivation. These differences were larger among men and mostly marked at birth for both sexes.

**Electronic supplementary material:**

The online version of this article (10.1186/s12889-019-6579-6) contains supplementary material, which is available to authorized users.

## Background

Life tables provide information on mortality rates and probabilities of death for specific populations defined by geographical regions and/or periods of time. They are important demographic tools as they are the basis for the estimation of life expectancy at birth, an important indicator of population health and development. Many factors are known to influence overall mortality, such as age, sex, geographical region, socioeconomic deprivation or ethnicity [1–4]. While the effect of, for example, age is largely unavoidable, the gap in mortality due to socioeconomic characteristics could be reduced with policies oriented to improve population living conditions and to change the social and economic structures [5].

Deprivation is a combination of individual and contextual parameters. Individual measures of deprivation are rarely available at population level and area-based measures are then used as a surrogate of individual measures even though they reflect the contextual deprivation. Many studies showed the existence of socioeconomic inequalities in health outcomes including mortality, either using measures of deprivation at individual level [6–8] or using area-based measures [9–11]. The inequalities can result from individual factors such as different lifestyle behaviours, namely, smoking, alcohol, physical activity and dietary habits, different health literacy or access to health care, among other factors. There can also be ecological deprivation inequalities, defined by varying levels of availability of services or measures of the wellbeing of the population of a given area.

Previous studies have found association between health outcomes and deprivation in Portugal [12, 13]. However, no life tables have been constructed by area-based socioeconomic level in Portugal yet. In fact, deprivation-specific life tables are available for very few European countries. An ecological measure of socioeconomic deprivation, the European Deprivation Index (EDI) [14, 15] has recently become available in Portugal. The first aim of this study was thus to build deprivation-specific life tables using the Portuguese version of the EDI. The second aim was to evaluate mortality ratios between deprivation groups and trends in inequalities between 2000 and 2002 and 2010–2012 in Portugal.

## Methods

### Socioeconomic deprivation

The Portuguese version of the European Deprivation Index was used as deprivation indicator. This index was built using a methodology first proposed by Pornet and colleagues in 2012 [16] and then applied to several European countries including Portugal [14, 15]. The index is based on census variables available for each country that are most associated with variables identified from the European Union Statistics on Income and Living Conditions (EU-SILC) survey [17]. The index for Portugal based on 2001 census includes percentage of: non-owned households, households without indoor flushing, residents with low education level (≤6th grade), household with 5 rooms or less, unemployed looking for a job, female residents aged 65 years or more, households without bath/shower and percentage of residents employed in manual occupations [15]. A score was obtained for each parish based on the census responses of its inhabitants. Most research on inequalities uses deprivation categorised according to quintiles. To build deprivation-specific life tables the EDI score was then categorized in five quintiles from the least deprived (q1) to the most deprived (q5) such that each quintile corresponded to 20% of the Portuguese population. Each deceased was assigned with the deprivation quintile corresponding to his/her parish of residence at the time of death.

### Mortality and population data

Mortality rates in life tables require counts of deaths (numerator of the rates) and population (denominator) stratified by demographic variables (age, sex, others). This information is usually made available by the national statistics offices. The number of deaths by sex, age group (0, 1–4, 5–9, …, 85+), year of death and area of residence (parish) was obtained by special request to the Statistics Portugal (*Instituto Nacional de Estatística*). As common practice, to increase estimates’ stability, three years of data were considered centred on each census year for which the life tables were estimated (2000–2002 and 2010–2012). Population data was retrieved from the Statistics Portugal website (www.ine.pt). Number of residents by sex, age group (0, 1–4, 5–9, …, 85+) and parish was only available for census years (2001, 2011) so that the population was considered constant over the three years of each studied period. There were 4241 parishes in Portugal in 2001, with a median population of 969 inhabitants (min-max: 39–81,845), while in 2011 this number increased to 4260 (median population: 892, min-max: 31–66,250).

In this study, both the numbers of deaths and people (residing in the parishes) were summed up across the parishes for each period by sex, age group and level of deprivation.

### Statistical analysis

When at subnational level, the counts of deaths and population produced by the national statistics offices are often available only by age groups (e.g. abridged) rather than by single years of age (e.g. complete) [18]. Several methods for building complete life tables from abridged data have been in use, namely, Elandt–Johnson, Kostaki, Brass logit, and Akima spline methods [19]. More recently, Rachet and colleagues [18] suggested a modelling approach to estimate smoothed mortality rates using flexible Poisson multivariable models. Death counts are modelled in the generalised linear model framework, considering a Poisson error and using splines to capture the effect of age. This method can use complete or abridged raw data allowing the estimation of complete life tables. This type of models was considered recommendable because it derives robust and unbiased estimates without making strong assumptions about age-specific mortality profiles. Also, a simulation study has shown that this method had better goodness of fit performance than other implemented methods [18]. The age-group specific death counts were here modelled within this generalised linear model framework, considering a Poisson error with a log link function. The offset was considered the person-years at risk. Male and female death counts were modelled separately. Covariates considered in the model were age (using the mid-age of each age group), quintile of deprivation (*dep*), period (2000–2002 vs 2010–2012) and interactions between deprivation and age, deprivation and period and period and age. The model can be written as:

$$ \log \left({d}_{age,i,j}\right)={\beta}_0+f(age)+\sum \limits_{i=2}^5{\beta}_{d_i}\cdotp { de p}_i+{g}_1\left( age\ast { de p}_i\right)+{\beta}_{per_j}\cdotp { per iod}_j+{g}_2\left( age\ast { per iod}_j\right)+\sum \limits_{k=2}^5{\beta}_{de{p_{per}}_k}\bullet { de p}_k\ast { per iod}_j+\log \left({pyrs}_{age,i,j}\right), $$

where *d*_*age,i,j*_ denotes the number of deaths and *pyrs*_*age,i,j*_ the number of person-years at risk for each age, deprivation group *i* and period *j*. The functions *f*, *g*_*1*_ and *g*_*2*_ represent restricted cubic splines. The knots positions were fixed a priori at ages 0, 1, 2 and 88 (for men) or 89 (for women). Although Rachet and colleagues considered further five knot positions selected from a set of 100 randomly simulated locations, here, we opted to consider knots at ages 10 to 50 at 10 years intervals since the other approach produced unrealistic predicted values. From these predefined knots position, the final number of knots was selected based on the Akaike Information Criterion (AIC).

Mortality rates were predicted from the fitted models by individual year of age (0–99), for each sex, period and quintile of deprivation. Life expectancies at birth were calculated from the fitted life tables. Mortality rate ratios (MRR) in terms of age were calculated from the predicted mortalities. MRR by EDI were calculated using the least deprived group as reference and the MRR by period using the period 2000–2002 as reference. The 95% confidence intervals (CI) for MRR were built assuming a normal distribution of log MRR and using the delta method. The derivations of the expressions for the CIs are presented as Additional file 1: Supplementary Material (S1).

All calculations were performed using STATA v13.1 and R v3.4.0. The STATA command *mvrs* was used for fitting the flexible Poisson model [20].

## Results

In the period 2000–2002 a total of 316,714 deaths were observed from which 219 (0.07%) were excluded due to unknown parish of residence at the time of death. In the period 2010–2012, the total number of deaths was 316,410 and only 75 deaths (0.02%) were excluded due to unknown parish or unknown age.

**Fig. 1:**
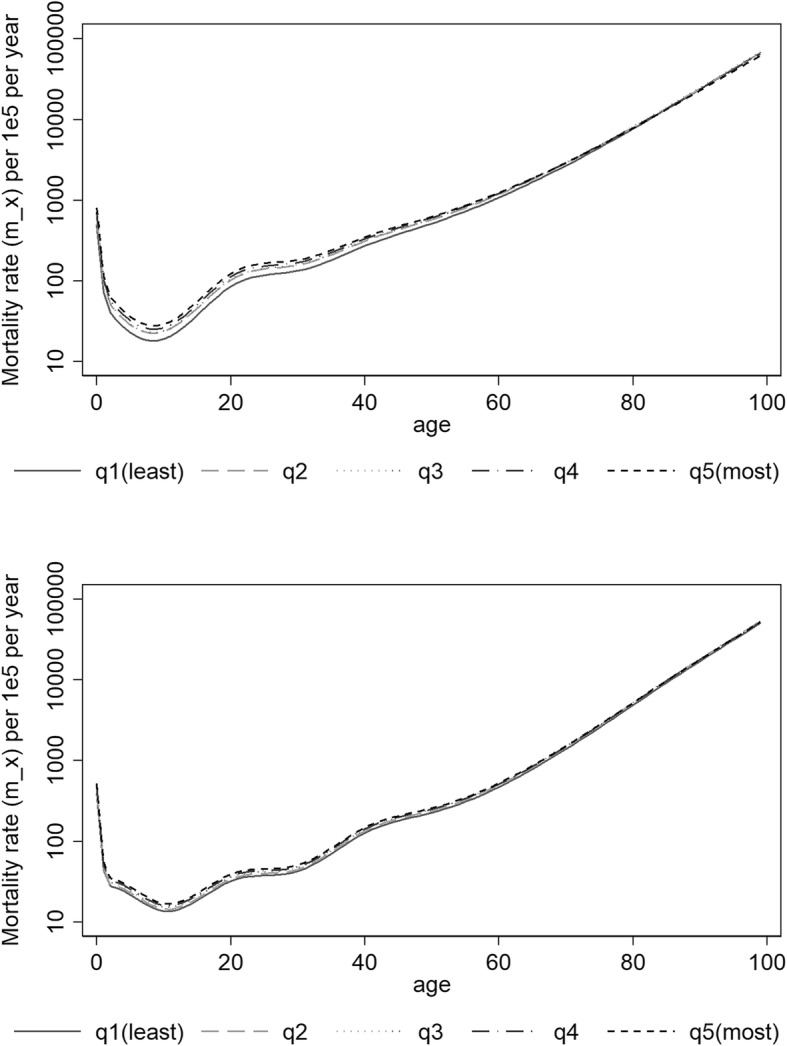
Predicted mortality rates (log scale) according to quintiles of socioeconomic deprivation 2000–2002 for Men (top) and Women (bottom)

**Fig. 2:**
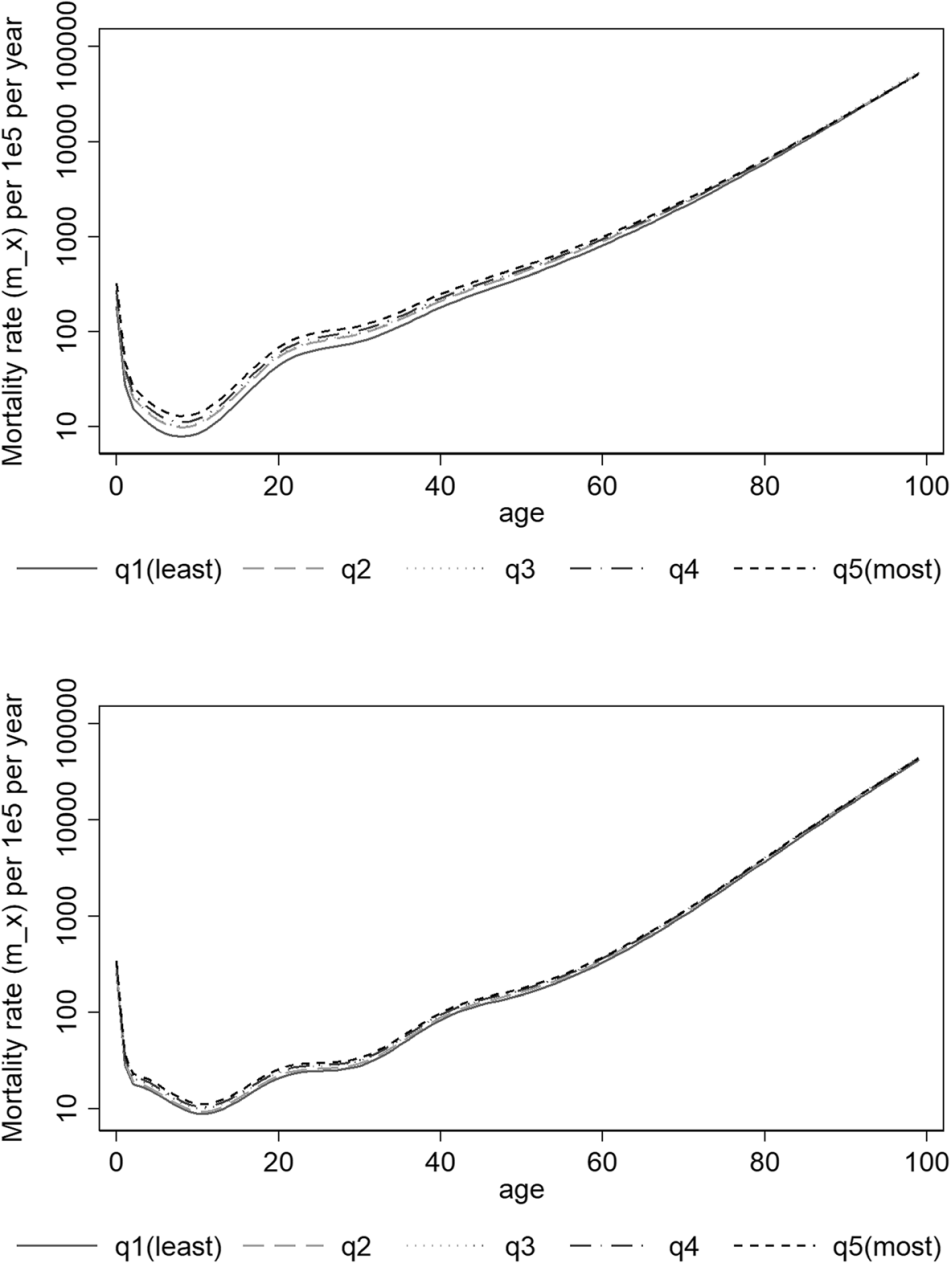
Predicted mortality rates (log scale) according to quintiles of socioeconomic deprivation 2010–2012 for Men (top) and Women (bottom)

**Fig. 3:**
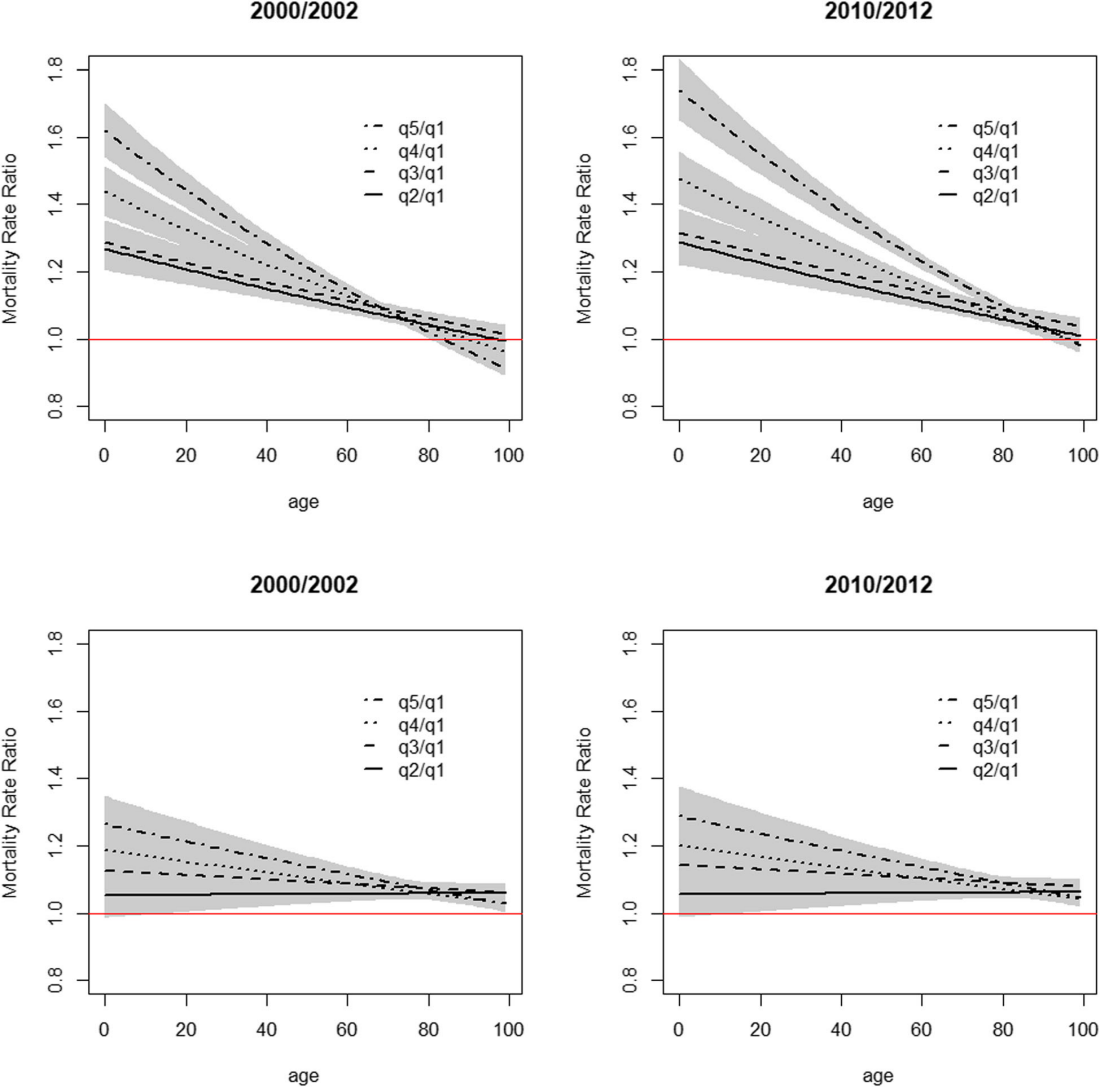
Mortality Rate Ratio as function of age between deprivation quintiles q2, q3, q4 and q5 and least deprived quintile (q1) for Men (top) and Women (bottom)

**Fig. 4:**
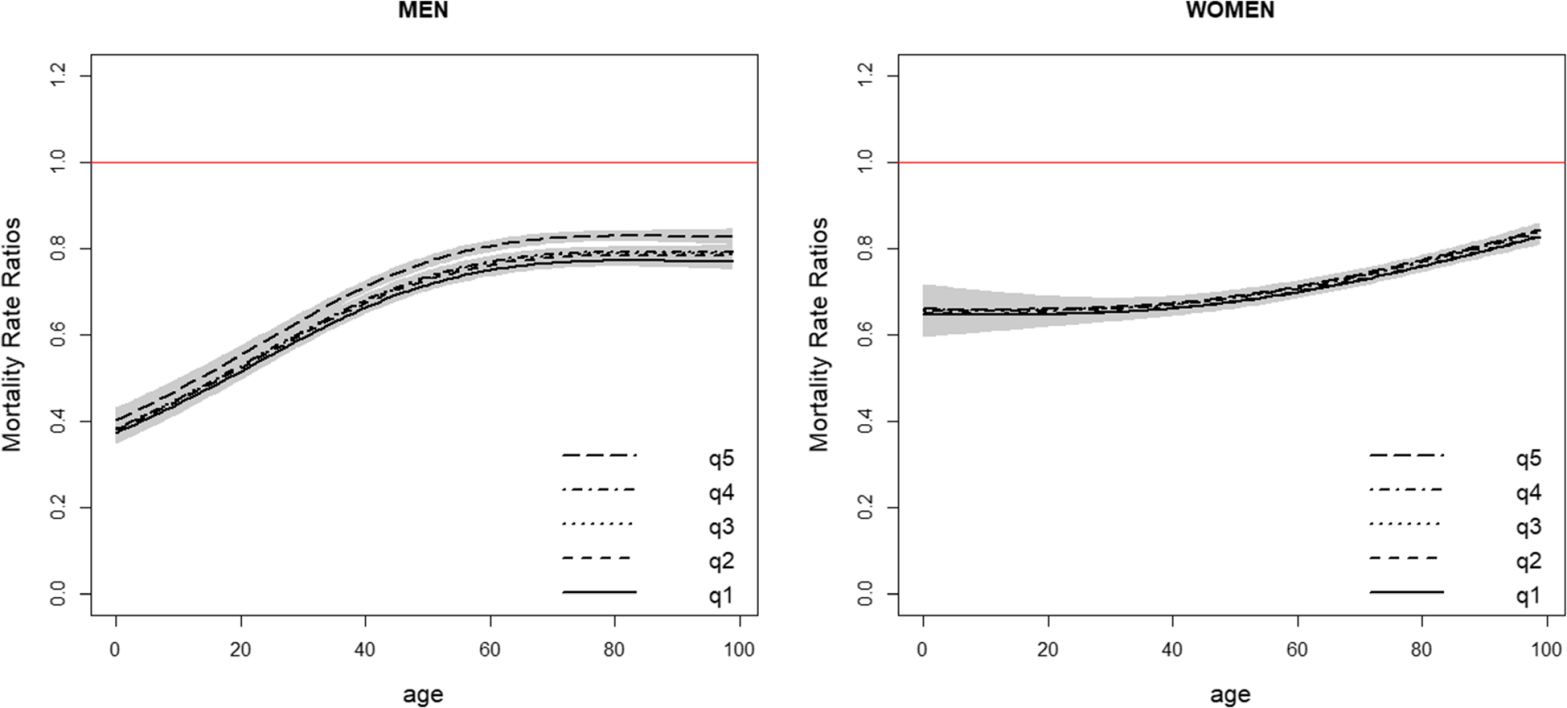
Mortality Rate Ratio as function of age between period 2010–2012 and 2000–2002 (reference) according to deprivation quintile for men (left) and women (right)

Both deprivation and period were found statistically significantly associated with mortality and all final fitted models included interactions between age and deprivation, period and age and period and deprivation, which were also found to be statistically significant. Higher mortality rates increasing with higher deprivation levels and decreasing with time periods were observed. The predicted mortality rates by age, sex, period and deprivation quintiles are presented in Additional file 2: Tables S1, Additional file 3: Table S2, Additional file 4: Table S3, Additional file 5: Table S4 and in Figs. 1 and 2. For all combinations period-sex-EDI analysed, the mortality rate first decreased with age reaching a minimum around 8–10 years-old and then steadily increased with age. For all deprivation quintiles q2 to q5, the mortality rates were in general significantly higher than the mortality rates of the least deprived group (q1) (Fig. 3). In men, at birth, the MRR between the most and the least deprived group was 1.62 (95%CI: 1.54–1.70) and 1.74 (95%CI: 1.65–1.83) in periods 2000–2002 and 2010–2012, respectively. In the first time period, the MRR’s decreased with age and from 81 onwards the ratio was no longer significantly different than 1. For the most recent period, this same pattern occurred from age 93 onwards (Fig. 3, top). In women, the MRRs were lower than the ones observed for men: at birth, the MRR between the two extreme deprivation groups was 1.26 (1.18–1.35) and 1.29 (1.20–1.38) in 2000–2002 and 2010–2012, respectively. The MRRs decreased with age but remained always significantly above one (Fig. 3, bottom). A reduction in the mortality rates was observed from the period 2000–2002 to the most recent period, 2010–2012. This reduction was significant for all ages (Fig. 4) and for both men and women. However, it was observed that the relative decrease in mortality was between 40 and 60% in less than 40 year olds but only 20% in older ages (over 60 year old) in men. This age gradient in the relative decrease between the two periods is less marked in women. For men, the reduction of mortality over time was less favourable for the most deprived group.

Life expectancy at birth increased from 74.0 years in 2000–2002 to 77.6 years in 2010–2012 in men and from 80.9 to 83.8 years in women. The gap in life expectancy at birth between the least and the most deprived group for men in the first period was 1.8 years. This gap slightly increased, over the ten-year period, to 2.1 years. For women, a smaller gap in life expectancy compared to men was observed. In 2000–2002 it was 1.0 year and it remained almost nearly constant over time (0.9 years in 2010–2012). The gap in life expectancy at 65 years was lower than at birth in both sexes. For men: 0.3 and 0.7 years for 2000–2002 and 2010–2012, respectively. In women, it was 0.5 years for both periods.

## Discussion

Persistent differences in mortality and life expectancy were observed according to ecological socioeconomic deprivation. These differences were larger among men and decreased with age for both sexes. Although mortality decreased significantly between the two periods, the deprivation gaps in mortality/life expectancy remained nearly constant from the period 2000–2002 to 2010–2012.

The smaller socioeconomic inequalities in mortality found in women have also been observed in other countries [21–23]. Several factors can contribute for this different pattern, including health behaviours and occupation. According to the last national health surveys made in Portugal, the prevalence of smoking was higher in men with low socioeconomic status while in women the prevalence was higher among individuals of high socioeconomic status [24]. Ribeiro and colleagues [25] found no influence of deprivation on longevity after 75 for men and a weak association for women in Portugal. We observed here a decrease in the mortality rate ratios between deprivation groups with age. Similarly to Ribeiro and colleagues findings, the difference between mortality rates ceased to be significant after ages around 80 years in men, while in women a slight but still significant difference remained at all ages.

Richardson and colleagues analysed the evolution in regional gap in life expectancy at birth from 1991 to 2008 within European Union countries [26]. No reduction in life expectancy gaps over the two decades analysed was observed, similarly to what has been observed in this study.

EUROSTAT (Statistical Office of the European Union) publishes estimates of life expectancy by age, sex and educational attainment level for several countries of the European Union including Portugal [27]. The difference in the estimates of life expectancy at birth between the two extreme education groups, “Less than primary”, “primary and lower secondary education” and “Tertiary education”, presents a high variability between countries. While this difference, for males in 2011, was 19.3 years in Czech Republic, it was as low as 3.6 years in Turkey. In Portugal, this gap was 4.5 years, the third lowest within the 17 EU countries with published information. For women, the gaps were generally lower, ranging from 1.7 (Italy and Malta) to 8.7 years (Bulgaria). In Portugal it was 2.0 years. Although the EUROSTAT results are based on a simple individual level deprivation measure while our study is based on a composite area-based indicator, it is interesting to note that relatively low gaps were found for Portugal. Similarly, in our study the gap in life expectancy observed was low (1–2 years). Much lower than, for instance, the observed for England where deprivation-specific life tables built using area-based indices are available [28]. Also, the smaller gap for women is in accordance with our findings.

The 2001 EDI version was used for both periods. By the time this study was developed the 2011 version was not available yet. However, preliminary results of the more recent version show that the EDI distribution by parish does not suffer major changes from 2001 to 2011. The impact of this limitation should therefore be small. For each individual, the EDI status was attributed based on the residence at the time of death. Although this can be a limitation, it is common practice in these types of studies and there was no information available that allowed matching mortality data for each individual to their residence history along their lifetime.

In this study we used a modelling approach to predict the mortality rate profiles by age. The flexible Poisson models have already been shown useful and valid to build life tables. They were used to build region-specific life tables within the CONCORD study (Cancer survival in five continents: a worldwide population-based study) [29] and by region, deprivation and ethnicity in England [4, 30]. The multivariable model allowed incorporating the deprivation and period effects, as well as interactions between them and with age, in a single model. Also it allowed obtaining complete life tables from abridged raw data.

Inference based on model predictions must have in consideration the correlation between the estimated parameters of the model. Ignoring this dependence and using the classical variance formulas as if the predicted values were observed ones would result in a sub estimation of the confidence intervals range. We thus derived and presented the variance estimators for the model-based mortality rate ratios taking into account this dependence.

This study is very relevant for the surveillance and monitoring of health inequalities, but it is important to highlight that these specific life tables are crucial tools to obtain reliable estimates of cancer survival within the relative survival data setting. In this setting, information on the cause of death is not available or not reliable. The disease-related survival (net survival) is then obtained indirectly by comparing the all-cause mortality of the cohort of patients with the mortality that would be experienced by individuals with the same demographic characteristics but free of the disease [31]. The information on this expected (also called background) mortality is obtained from population life tables, assuming that the mortality due to the disease in question is negligible relatively to the overall mortality [32]. To obtain valid net survival estimates, the population mortality should correctly reflect the expected mortality for each patient. The use of general life tables in the estimation of net survival for subgroups of the population with different overall mortality can lead to biased estimates of net survival. Estimation of net survival by deprivation is a situation where the use of general life tables can lead to overestimation of net survival in affluent groups, if these groups have a lower overall mortality than the general population, and the underestimation of net survival in the deprived groups, if these groups have a higher mortality than the general population [33, 34]. These questions arise also when stratifying by other factors that can influence overall mortality such as ethnicity [35].

## Conclusion

In conclusion, this study has shown the existence of persistent socioeconomic inequalities in overall mortality in Portugal. Deprivation-specific life tables were built for Portugal. These life tables can therefore be used for monitoring inequalities and in future studies that require background mortality information in the estimation of deprivation-specific net survival from any specific disease.

## Additional files

Additional file 1: **Supplementary S1.** Estimation of confidence intervals for model-based predicted mortality rate ratios. (PDF 111 kb)

Additional file 2: **Table S1.** Life tables by deprivation quintile for men in the period 2000–2002. (PDF 411 kb)

Additional file 3: **Table S2.** Life tables by deprivation quintile for women in the period 2000–2002. (PDF 411 kb)

Additional file 4: **Table S3.** Life tables by deprivation quintile for men in the period 2010–2012. (PDF 411 kb)

Additional file 5: **Table S4.** Life tables by deprivation quintile for women in the period 2010–2012. (PDF 411 kb)

## Abbreviations

AIC: Akaike Information Criterion
CI: Confidence interval
EDI: European Deprivation Index
EUROSTAT: Statistical Office of the European Union
EU-SILC: European Union Statistics on Income and Living Conditions
MRR: Mortality Rate Ratio
