# Supplementary material for: Deprivation-specific life tables using multivariable flexible modelling – trends from 2000–2002 to 2010–2012, Portugal
Source: BMC Public Health. 2019 Mar 7;19:276. doi: 10.1186/s12889-019-6579-6 (PMC6407195; doi:10.1186/s12889-019-6579-6)
Supplement: Supplementary file 1 — Supplementary S1. Estimation of confidence intervals for model-based predicted mortality rate ratios. (PDF 111 kb) [file 12889_2019_6579_MOESM1_ESM.pdf]

Deprivation-specific life tables using multivariables flexible modelling - trends from 2001 to 2011, Portugal

Luis Antunes, Denisa Mendonça, Ana Isabel Ribeiro, Camille Maringe, Bernard Rachet

Supplementary Material S1

Estimation of confidence intervals for model-based predicted mortality rate ratios

Considering a GLM model with Poisson error for the number of deaths by age, deprivation quintile and period:

$$log(d_{age,i,j}) = \beta_0 + f(age) + \sum_{k=2}^{5} \beta_{d_k} \cdot dep_k + g_1(age * dep_i) + \beta_{period_j} \cdot period_j +$$
$$+ \sum_{k=2}^{5} \beta_{dep\_period_k} \cdot period_j * dep_k + g_2(age * period_j) + log(pyrs_{age,i,j})$$

Reference categories:

Deprivation: first quintile (least deprived)

Period: 2000-02

Considering linear interaction between age and deprivation:

$$g_1(age * dep_i) = \sum_{i=2}^{5} \beta_{age\_dep_i} \cdot age * dep_i$$

Considering interaction of period with splines for age with 1 internal knot:

$$g_2(age * period_j) = \beta_{period\_age_1} \cdot (period_j * age)_S + \beta_{period\_age_2} \cdot v(period_j * age),$$

where $(period_j * age)_S$ represents the standardised $period * age$ variable and $v(period_j * age)$ represents the orthogonalised spline basis.

Mortality rate:

$$log(R_{age,i,j}) = \beta_0 + f(age) + \sum_{k=2}^{5} \beta_{d_k} \cdot dep_k + g_1(age * dep_i) + \beta_{period_j} \cdot period_j +$$
$$+ \sum_{k=2}^{5} \beta_{dep\_period_k} \cdot period_j * dep_i + g_2(age * period_j)$$

where

$$log(R_{age,i,j}) = log(d_{age,i,j}) - log(pyrs_{age,i,j}) = log\left(\frac{d_{age,i,j}}{pyrs_{age,i,j}}\right)$$

Mortality rate ratio between two different deprivation groups, same period:

$$log\left(\frac{R_{age,i=a,j}}{R_{age,i=b,j}}\right) =$$

$$= \beta_0 + f(age) + \beta_{d_{i=a}} + \beta_{age\_dep=a} \cdot age + \beta_{period_j} \cdot period_j + \beta_{dep\_period=a} \cdot period_j +$$

$$+ g_2(age * period_j) -$$

$$[\beta_0 + f(age) + \beta_{d_{i=b}} + \beta_{age\_dep=b} \cdot age + \beta_{period_j} \cdot period_j + \beta_{dep\_period=b} \cdot period_j +$$

$$+ g_2(age * period_j)] =$$

$$= \beta_{d_{i=a}} + \beta_{age\_dep=a} \cdot age + \beta_{dep\_period=a} \cdot period_j - (\beta_{d_{i=b}} + \beta_{age\_dep=b} \cdot age + \beta_{dep\_period=b} \cdot period_j)$$

$$= (\beta_{d_{i=a}} - \beta_{d_{i=b}}) + (\beta_{age\_dep=a} - \beta_{age\_dep=b}) \cdot age + (\beta_{dep\_period=a} - \beta_{dep\_period=b}) \cdot period_j$$

Calculate Confidence Interval assuming normality of $log(RR)$.

The variance of the $log(RR)$ can be estimated using the delta method:

$$VAR[log(RR)(\beta)] \simeq \left[\frac{\partial log(RR)}{\partial \beta}\right]_{\beta=\hat{\beta}} \times VAR[\hat{\beta}] \times \left[\frac{\partial log(RR)}{\partial \beta}\right]^T_{\beta=\hat{\beta}}$$

$$\left[\frac{\partial log(RR)}{\partial \beta}\right]_{\beta=\hat{\beta}} = \begin{bmatrix} 1 & -1 & age & -age & period & -period \end{bmatrix}$$

$$VAR[\hat{\beta}] = \begin{bmatrix} VAR[\beta_{d_{i=a}}] & COV[\beta_{d_{i=a}}, \beta_{d_{i=b}}] & \cdot & \cdot & \cdot & \cdot \\ \cdot & VAR[\beta_{d_{i=b}}] & \cdot & \cdot & \cdot & \cdot \\ \cdot & \cdot & \cdot & \cdot & \cdot & \cdot \\ \cdot & \cdot & \cdot & \cdot & \cdot & \cdot \\ \cdot & \cdot & \cdot & \cdot & \cdot & \cdot \\ \cdot & \cdot & \cdot & \cdot & COV[\beta_{dep\_period=b}, \beta_{dep\_period=a}] & VAR[\beta_{dep\_period=b}] \end{bmatrix}$$

The 95% confidence interval for the mortality rate ratio is then given by:

$$exp\left[log\left(\frac{R_{age,i=a,j}}{R_{age,i=b,j}}\right) \pm 1.96 \times \sqrt{VAR[log(RR)]}\right]$$

Considering that the deprivation group $i = b$ is the reference group, the expressions above simplify to:

$$log\left(\frac{R_{age,i=a,j}}{R_{age,i=b,j}}\right) =$$
$$= \beta_{d_{i=a}} + \beta_{age\_dep=a} \cdot age + \beta_{dep\_period=a} \cdot period_j$$

$$\left[\frac{\partial log(RR)}{\partial \beta}\right]_{\beta=\hat{\beta}} = \begin{bmatrix} 1 & age & period \end{bmatrix}$$

$$VAR[\hat{\beta}] = \begin{bmatrix} VAR[\beta_{d_{i=a}}] & COV[\beta_{d_{i=a}}, \beta_{age\_dep=a}] & \cdot \\ \cdot & VAR[\beta_{age\_dep=a}] & \cdot \\ \cdot & COV[\beta_{age\_dep=a}, \beta_{dep\_period=a}] & VAR[\beta_{dep\_period=a}] \end{bmatrix}$$

Mortality rate ratio between two different periods ($period = 1/period = 0$), same deprivation group:

$$log\left(\frac{R_{age,i,j=1}}{R_{age,i,j=0}}\right) =$$
$$= \beta_0 + f(age) + \beta_{d_i} + \beta_{age\_dep=i} + \beta_{period_1} + \beta_{dep\_period=i1} + g_2(age * period_1) -$$
$$- (\beta_0 + f(age) + \beta_{d_i} + \beta_{age\_dep=i} + g_2(age * period_0)) =$$
$$= \beta_{period_1} + \beta_{dep\_period=i1} + g_2(age * period_1) - g_2(age * period_0)$$

Assuming only one knot for the interaction age*period as stated above:

$$log\left(\frac{R_{age,i,j=1}}{R_{age,i,j=0}}\right) = \beta_{period_1} + \beta_{dep\_period=i1} + \beta_{period\_age_1} \cdot ((period_1 * age)_S - (period_0 * age)_S) +$$
$$+ \beta_{period\_age_2} \cdot (v(period_1 * age) - v(period_0 * age))$$

Again, the variance of the $log(RR)$ can be estimated using the delta method:

$$VAR[log(RR)(\beta)] \simeq \left[\frac{\partial log(RR)}{\partial \beta}\right]_{\beta=\hat{\beta}} \times VAR[\hat{\beta}] \times \left[\frac{\partial log(RR)}{\partial \beta}\right]^T_{\beta=\hat{\beta}}$$

$$\left[\frac{\partial log(RR)}{\partial \beta}\right]_{\beta=\hat{\beta}} = \begin{bmatrix} 1 & 1 & (period_1 * age)_S - (period_0 * age)_S & v(period_1 * age) - v(period_0 * age) \end{bmatrix}$$

$$VAR[\hat{\beta}] = \begin{bmatrix} VAR[\beta_{period_1}] & COV[\beta_{period_1}, \beta_{dep\_period=i1}] & \cdot & \cdot \\ \cdot & VAR[\beta_{dep\_period=i1}] & \cdot & \cdot \\ \cdot & \cdot & \cdot & \cdot \\ \cdot & \cdot & COV[\beta_{period\_age_2}, \beta_{period\_age_1}] & VAR[\beta_{period\_age_2}] \end{bmatrix}$$

The 95% confidence interval for the mortality rate ratio is then given by:

$$exp\left[log\left(\frac{R_{age,i,j=1}}{R_{age,i,j=0}}\right) \pm 1.96 \times \sqrt{VAR[log(RR)]}\right]$$
