# Supplementary material for: Deprivation-specific life tables using multivariable flexible modelling – trends from 2000–2002 to 2010–2012, Portugal
Source: BMC Public Health. 2019 Mar 7;19:276. doi: 10.1186/s12889-019-6579-6 (PMC6407195; doi:10.1186/s12889-019-6579-6)
Supplement: Supplementary file 2 — Table S1. Life tables by deprivation quintile for men in the period 2000–2002. (PDF 411 kb) [file 12889_2019_6579_MOESM2_ESM.pdf]

Table S1 - Life tables by deprivation quintile (1-Least deprived) for men in the period 2000-2002 (m_x - mortality rate; e_x - life expectancy at age x).

age	EDI = 1		EDI = 2		EDI = 3		EDI = 4		EDI = 5	
	m_x	e_x	m_x	e_x	m_x	e_x	m_x	e_x	m_x	e_x
0	496,1	75,1	628,8	74,0	638,1	73,8	712,8	73,6	802,9	73,3
1	72,8	74,5	92,0	73,5	93,4	73,3	104,2	73,1	117,1	72,9
2	40,5	73,5	51,0	72,5	51,8	72,3	57,7	72,2	64,7	72,0
3	32,3	72,6	40,6	71,6	41,2	71,4	45,8	71,2	51,4	71,0
4	26,7	71,6	33,5	70,6	34,0	70,4	37,8	70,3	42,3	70,1
5	23,0	70,6	28,8	69,6	29,2	69,4	32,3	69,3	36,1	69,1
6	20,5	69,6	25,6	68,7	26,0	68,5	28,7	68,3	32,0	68,1
7	19,0	68,6	23,7	67,7	24,0	67,5	26,5	67,3	29,5	67,1
8	18,3	67,7	22,8	66,7	23,1	66,5	25,5	66,4	28,3	66,2
9	18,4	66,7	22,8	65,7	23,1	65,5	25,5	65,4	28,2	65,2
10	19,2	65,7	23,7	64,7	24,1	64,5	26,5	64,4	29,3	64,2
11	20,8	64,7	25,7	63,7	26,1	63,5	28,6	63,4	31,6	63,2
12	23,4	63,7	28,8	62,8	29,2	62,5	32,0	62,4	35,3	62,2
13	27,0	62,7	33,1	61,8	33,6	61,6	36,8	61,5	40,5	61,3
14	31,7	61,7	38,9	60,8	39,5	60,6	43,1	60,5	47,4	60,3
15	37,8	60,8	46,2	59,8	46,9	59,6	51,1	59,5	56,1	59,3
16	45,3	59,8	55,2	58,8	56,1	58,6	61,0	58,5	66,8	58,4
17	54,2	58,8	65,9	57,9	66,9	57,7	72,7	57,6	79,5	57,4
18	64,3	57,8	78,0	56,9	79,2	56,7	85,9	56,6	93,8	56,4
19	75,2	56,9	91,0	56,0	92,4	55,8	100,0	55,7	109,1	55,5
20	86,0	55,9	103,7	55,0	105,4	54,8	113,9	54,7	124,0	54,5
21	95,6	55,0	115,1	54,1	117,0	53,9	126,2	53,8	137,1	53,6
22	103,8	54,0	124,6	53,1	126,6	52,9	136,4	52,8	147,9	52,7
23	110,3	53,1	132,1	52,2	134,3	52,0	144,4	51,9	156,3	51,8
24	115,3	52,1	137,8	51,3	140,0	51,1	150,3	51,0	162,5	50,8
25	119,0	51,2	141,9	50,3	144,2	50,1	154,5	50,1	166,8	49,9
26	121,9	50,3	144,9	49,4	147,3	49,2	157,6	49,1	169,8	49,0
27	124,2	49,3	147,4	48,5	149,8	48,3	160,0	48,2	172,1	48,1
28	126,7	48,4	149,9	47,5	152,4	47,3	162,5	47,3	174,4	47,2
29	129,7	47,4	153,1	46,6	155,6	46,4	165,7	46,4	177,6	46,3
30	133,9	46,5	157,7	45,7	160,3	45,5	170,4	45,4	182,3	45,3
31	139,9	45,6	164,3	44,8	167,0	44,6	177,2	44,5	189,3	44,4
32	147,7	44,6	173,0	43,8	175,9	43,6	186,3	43,6	198,7	43,5
33	157,3	43,7	183,9	42,9	187,0	42,7	197,7	42,7	210,5	42,6
34	168,9	42,8	196,9	42,0	200,2	41,8	211,3	41,8	224,6	41,7
35	182,2	41,8	212,0	41,1	215,5	40,9	227,2	40,9	241,0	40,8
36	197,5	40,9	229,1	40,1	233,0	40,0	245,1	39,9	259,6	39,9
37	214,4	40,0	248,2	39,2	252,4	39,1	265,1	39,0	280,3	39,0
38	232,9	39,1	268,9	38,3	273,5	38,2	286,8	38,1	302,7	38,1
39	252,7	38,2	291,0	37,4	296,0	37,3	309,9	37,2	326,5	37,2
40	273,3	37,2	314,0	36,5	319,4	36,4	333,8	36,4	351,1	36,3
41	294,3	36,4	337,3	35,7	343,1	35,5	358,0	35,5	376,0	35,4
42	315,7	35,5	360,9	34,8	367,1	34,6	382,4	34,6	400,9	34,6
43	337,5	34,6	384,9	33,9	391,5	33,7	407,2	33,7	426,1	33,7
44	359,8	33,7	409,3	33,0	416,4	32,9	432,3	32,9	451,7	32,8
45	382,8	32,8	434,5	32,2	442,0	32,0	458,1	32,0	477,8	32,0
46	406,8	31,9	460,5	31,3	468,6	31,1	484,9	31,2	504,8	31,1
47	432,0	31,1	487,9	30,4	496,4	30,3	512,9	30,3	533,1	30,3
48	458,9	30,2	517,0	29,6	526,0	29,4	542,5	29,5	562,9	29,5
49	487,8	29,3	548,2	28,7	557,8	28,6	574,4	28,6	595,0	28,6

Table S1 (cont.) - Life tables by deprivation quintile (1-Least deprived) for men in the period 2000-2002 (m_x - mortality rate; e_x - life expectancy at age x).

age	EDI = 1		EDI = 2		EDI = 3		EDI = 4		EDI = 5	
	m_x	e_x	m_x	e_x	m_x	e_x	m_x	e_x	m_x	e_x
50	519,3	28,5	582,2	27,9	592,4	27,7	609,0	27,8	629,7	27,8
51	554,0	27,6	619,5	27,1	630,4	26,9	647,0	26,9	667,9	27,0
52	592,2	26,8	660,7	26,2	672,4	26,1	688,9	26,1	709,9	26,1
53	634,4	25,9	706,0	25,4	718,6	25,2	735,0	25,3	756,2	25,3
54	681,1	25,1	756,1	24,6	769,6	24,4	785,8	24,5	807,1	24,5
55	732,6	24,2	811,3	23,8	825,8	23,6	841,9	23,7	863,2	23,7
56	789,6	23,4	872,3	22,9	887,9	22,8	903,7	22,9	925,0	22,9
57	852,7	22,6	939,6	22,1	956,6	22,0	971,9	22,1	993,1	22,1
58	922,6	21,8	1014,1	21,3	1032,4	21,2	1047,3	21,3	1068,3	21,3
59	1000,0	21,0	1096,5	20,6	1116,4	20,4	1130,5	20,5	1151,2	20,6
60	1085,8	20,2	1187,7	19,8	1209,3	19,6	1222,6	19,7	1242,9	19,8
61	1181,1	19,4	1288,8	19,0	1312,3	18,9	1324,5	19,0	1344,2	19,0
62	1287,0	18,6	1400,9	18,2	1426,5	18,1	1437,4	18,2	1456,3	18,3
63	1404,7	17,9	1525,3	17,5	1553,3	17,4	1562,5	17,5	1580,3	17,5
64	1535,7	17,1	1663,4	16,8	1694,0	16,6	1701,3	16,7	1717,7	16,8
65	1681,5	16,4	1816,9	16,0	1850,4	15,9	1855,3	16,0	1870,0	16,1
66	1844,1	15,6	1987,6	15,3	2024,4	15,2	2026,3	15,3	2038,9	15,4
67	2025,3	14,9	2177,6	14,6	2218,1	14,5	2216,5	14,6	2226,5	14,7
68	2227,7	14,2	2389,3	13,9	2433,9	13,8	2428,1	13,9	2434,8	14,0
69	2453,7	13,5	2625,3	13,2	2674,4	13,1	2663,6	13,2	2666,4	13,3
70	2706,4	12,8	2888,5	12,6	2942,7	12,5	2925,9	12,6	2924,0	12,7
71	2989,0	12,2	3182,3	11,9	3242,2	11,8	3218,4	12,0	3210,8	12,1
72	3305,4	11,5	3510,5	11,3	3576,8	11,2	3544,6	11,3	3530,2	11,4
73	3659,8	10,9	3877,4	10,7	3950,8	10,6	3908,8	10,7	3886,2	10,8
74	4057,1	10,3	4287,8	10,1	4369,2	10,0	4315,5	10,1	4283,3	10,2
75	4502,8	9,7	4747,1	9,5	4837,5	9,4	4770,1	9,5	4726,4	9,7
76	5002,9	9,1	5261,4	9,0	5361,9	8,9	5278,4	9,0	5221,2	9,1
77	5564,6	8,6	5837,7	8,4	5949,5	8,3	5847,2	8,5	5773,9	8,6
78	6195,6	8,0	6483,8	7,9	6608,4	7,8	6483,9	7,9	6391,6	8,0
79	6905,0	7,5	7208,5	7,4	7347,4	7,3	7197,0	7,4	7082,5	7,5
80	7703,0	7,0	8021,7	6,9	8176,8	6,8	7996,1	6,9	7855,4	7,1
81	8600,8	6,5	8934,7	6,4	9107,9	6,4	8891,8	6,5	8720,5	6,6
82	9611,5	6,1	9960,2	6,0	10153,8	5,9	9896,4	6,0	9689,2	6,2
83	10749,7	5,6	11112,4	5,6	11329,0	5,5	11023,5	5,6	10774,2	5,7
84	12032,0	5,2	12407,4	5,2	12650,0	5,1	12288,4	5,2	11990,0	5,3
85	13477,0	4,8	13863,4	4,8	14135,3	4,7	13708,4	4,8	13352,6	4,9
86	15105,9	4,5	15500,8	4,4	15805,7	4,3	15302,9	4,5	14880,3	4,6
87	16942,6	4,1	17342,8	4,1	17685,0	4,0	17093,9	4,1	16593,4	4,2
88	19014,0	3,8	19415,3	3,7	19799,5	3,7	19105,9	3,8	18514,9	3,9
89	21348,6	3,5	21745,7	3,4	22177,2	3,4	21364,8	3,5	20668,5	3,6
90	23972,1	3,2	24358,1	3,1	24842,8	3,1	23892,9	3,2	23074,8	3,3
91	26918,0	2,9	27284,3	2,9	27828,8	2,8	26720,3	2,9	25761,3	3,0
92	30226,0	2,6	30562,1	2,6	31173,7	2,6	29882,2	2,7	28760,5	2,7
93	33940,4	2,4	34233,6	2,4	34920,7	2,3	33418,2	2,4	32108,9	2,5
94	38111,3	2,2	38346,1	2,1	39118,0	2,1	37372,7	2,2	35847,2	2,3
95	42794,8	1,9	42952,8	1,9	43819,8	1,9	41795,2	2,0	40020,6	2,0
96	48053,8	1,7	48112,8	1,7	49086,8	1,7	46741,0	1,7	44679,9	1,8
97	53959,1	1,4	53892,8	1,4	54986,8	1,4	52272,0	1,4	49881,7	1,5
98	60590,1	1,0	60367,1	1,0	61596,0	1,0	58457,5	1,1	55689,1	1,1
99	68036,0	0,5	67619,1	0,5	68999,6	0,5	65375,0	0,5	62172,7	0,5
