# Supplementary material for: Deprivation-specific life tables using multivariable flexible modelling – trends from 2000–2002 to 2010–2012, Portugal
Source: BMC Public Health. 2019 Mar 7;19:276. doi: 10.1186/s12889-019-6579-6 (PMC6407195; doi:10.1186/s12889-019-6579-6)
Supplement: Supplementary file 3 — Table S2. Life tables by deprivation quintile for women in the period 2000–2002. (PDF 411 kb) [file 12889_2019_6579_MOESM3_ESM.pdf]

Table S2 - Life tables by deprivation quintile (1-Least deprived) for women in the period 2000-2002 (m_x - mortality rate; e_x - life expectancy at age x).

age	EDI = 1		EDI = 2		EDI = 3		EDI = 4		EDI = 5	
	m_x	e_x	m_x	e_x	m_x	e_x	m_x	e_x	m_x	e_x
0	407,7	81,6	429,3	81,0	458,9	80,8	482,9	80,8	515,3	80,6
1	44,1	80,9	46,4	80,4	49,6	80,2	52,1	80,2	55,6	80,0
2	27,8	79,9	29,3	79,4	31,3	79,2	32,9	79,3	35,0	79,1
3	26,5	78,9	27,9	78,4	29,7	78,2	31,2	78,3	33,2	78,1
4	24,3	78,0	25,6	77,5	27,3	77,3	28,7	77,3	30,5	77,1
5	21,9	77,0	23,0	76,5	24,6	76,3	25,7	76,3	27,4	76,2
6	19,5	76,0	20,5	75,5	21,8	75,3	22,9	75,3	24,3	75,2
7	17,3	75,0	18,3	74,5	19,4	74,3	20,3	74,4	21,6	74,2
8	15,6	74,0	16,5	73,5	17,5	73,3	18,3	73,4	19,4	73,2
9	14,4	73,0	15,2	72,5	16,2	72,3	16,9	72,4	17,9	72,2
10	13,8	72,0	14,5	71,5	15,4	71,4	16,1	71,4	17,1	71,2
11	13,7	71,1	14,5	70,6	15,4	70,4	16,0	70,4	17,0	70,2
12	14,2	70,1	15,0	69,6	15,9	69,4	16,6	69,4	17,6	69,3
13	15,2	69,1	16,1	68,6	17,0	68,4	17,7	68,4	18,7	68,3
14	16,7	68,1	17,6	67,6	18,6	67,4	19,4	67,5	20,5	67,3
15	18,6	67,1	19,6	66,6	20,8	66,4	21,6	66,5	22,8	66,3
16	21,0	66,1	22,1	65,6	23,4	65,4	24,3	65,5	25,6	65,3
17	23,7	65,1	25,0	64,6	26,4	64,4	27,4	64,5	28,9	64,3
18	26,7	64,1	28,1	63,6	29,7	63,5	30,8	63,5	32,5	63,3
19	29,7	63,2	31,3	62,7	33,0	62,5	34,2	62,5	36,1	62,4
20	32,4	62,2	34,2	61,7	36,1	61,5	37,3	61,6	39,3	61,4
21	34,6	61,2	36,5	60,7	38,4	60,5	39,8	60,6	41,8	60,4
22	36,1	60,2	38,1	59,7	40,1	59,5	41,5	59,6	43,6	59,4
23	37,1	59,2	39,2	58,7	41,2	58,6	42,6	58,6	44,7	58,5
24	37,7	58,3	39,8	57,8	41,9	57,6	43,2	57,6	45,4	57,5
25	38,1	57,3	40,2	56,8	42,3	56,6	43,6	56,7	45,7	56,5
26	38,4	56,3	40,5	55,8	42,6	55,6	43,9	55,7	46,0	55,5
27	38,9	55,3	41,0	54,8	43,0	54,7	44,3	54,7	46,4	54,6
28	39,6	54,3	41,8	53,9	43,8	53,7	45,1	53,7	47,2	53,6
29	40,9	53,4	43,1	52,9	45,2	52,7	46,5	52,8	48,6	52,6
30	42,9	52,4	45,3	51,9	47,5	51,7	48,7	51,8	50,9	51,6
31	46,0	51,4	48,6	50,9	50,9	50,8	52,2	50,8	54,5	50,7
32	50,3	50,4	53,0	50,0	55,5	49,8	56,9	49,8	59,4	49,7
33	55,7	49,5	58,8	49,0	61,5	48,8	63,0	48,9	65,7	48,7
34	62,5	48,5	65,9	48,0	68,9	47,8	70,5	47,9	73,5	47,8
35	70,6	47,5	74,5	47,0	77,8	46,9	79,5	46,9	82,9	46,8
36	80,0	46,6	84,4	46,1	88,1	45,9	90,0	46,0	93,8	45,8
37	90,7	45,6	95,7	45,1	99,9	44,9	101,9	45,0	106,1	44,9
38	102,5	44,6	108,2	44,2	112,8	44,0	115,0	44,1	119,6	43,9
39	115,0	43,7	121,4	43,2	126,4	43,0	128,8	43,1	133,9	43,0
40	127,6	42,7	134,7	42,3	140,2	42,1	142,8	42,2	148,3	42,0
41	139,6	41,8	147,4	41,3	153,4	41,2	156,1	41,2	162,0	41,1
42	150,9	40,8	159,4	40,4	165,7	40,2	168,4	40,3	174,7	40,2
43	161,4	39,9	170,4	39,4	177,1	39,3	179,9	39,4	186,5	39,2
44	171,2	39,0	180,7	38,5	187,7	38,4	190,5	38,4	197,3	38,3
45	180,3	38,0	190,4	37,6	197,5	37,4	200,3	37,5	207,4	37,4
46	189,0	37,1	199,6	36,6	207,0	36,5	209,7	36,6	216,9	36,4
47	197,6	36,2	208,7	35,7	216,2	35,6	218,9	35,6	226,3	35,5
48	206,4	35,2	218,0	34,8	225,8	34,6	228,4	34,7	236,0	34,6
49	215,9	34,3	228,1	33,9	236,0	33,7	238,6	33,8	246,3	33,7

Table S2 (cont.) - Life tables by deprivation quintile (1-Least deprived) for women in the period 2000-2002 (m_x - mortality rate; e_x - life expectancy at age x).

age	EDI = 1		EDI = 2		EDI = 3		EDI = 4		EDI = 5	
	m_x	e_x	m_x	e_x	m_x	e_x	m_x	e_x	m_x	e_x
50	226,6	33,4	239,4	32,9	247,6	32,8	250,0	32,9	257,9	32,8
51	239,0	32,5	252,4	32,0	260,9	31,9	263,3	32,0	271,4	31,9
52	253,2	31,5	267,5	31,1	276,3	31,0	278,6	31,0	287,1	30,9
53	269,7	30,6	285,0	30,2	294,1	30,0	296,3	30,1	305,1	30,0
54	288,6	29,7	304,9	29,3	314,5	29,1	316,6	29,2	325,7	29,1
55	310,2	28,8	327,7	28,3	337,8	28,2	339,8	28,3	349,4	28,2
56	334,8	27,9	353,8	27,4	364,5	27,3	366,3	27,4	376,4	27,3
57	362,9	27,0	383,5	26,5	394,8	26,4	396,4	26,5	407,1	26,4
58	394,9	26,1	417,4	25,6	429,4	25,5	430,8	25,6	442,1	25,5
59	431,4	25,2	456,0	24,7	468,8	24,6	470,0	24,7	482,0	24,6
60	473,0	24,3	500,0	23,9	513,7	23,7	514,6	23,8	527,3	23,7
61	520,4	23,4	550,2	23,0	564,9	22,9	565,3	23,0	579,0	22,9
62	574,5	22,5	607,3	22,1	623,1	22,0	623,1	22,1	637,7	22,0
63	636,1	21,6	672,5	21,2	689,6	21,1	689,0	21,2	704,7	21,1
64	706,4	20,8	746,9	20,4	765,3	20,3	764,1	20,4	780,9	20,3
65	786,6	19,9	831,8	19,5	851,8	19,4	849,7	19,5	867,8	19,4
66	878,3	19,0	928,7	18,7	950,4	18,6	947,3	18,7	966,9	18,6
67	983,0	18,2	1039,5	17,8	1063,1	17,7	1058,7	17,8	1079,9	17,8
68	1102,6	17,4	1166,1	17,0	1191,8	16,9	1185,9	17,0	1208,8	17,0
69	1239,4	16,6	1310,8	16,2	1338,8	16,1	1331,1	16,2	1355,8	16,2
70	1395,7	15,8	1476,3	15,4	1506,8	15,3	1496,9	15,4	1523,7	15,4
71	1574,5	15,0	1665,5	14,6	1698,8	14,6	1686,2	14,7	1715,3	14,6
72	1779,0	14,2	1881,9	13,9	1918,2	13,8	1902,5	13,9	1934,0	13,8
73	2012,7	13,5	2129,3	13,1	2169,0	13,1	2149,4	13,2	2183,5	13,1
74	2279,9	12,7	2412,2	12,4	2455,5	12,3	2431,3	12,4	2468,3	12,4
75	2585,3	12,0	2735,4	11,7	2782,7	11,6	2753,0	11,7	2793,0	11,7
76	2934,0	11,3	3104,6	11,0	3156,2	10,9	3120,0	11,0	3163,1	11,0
77	3332,0	10,6	3526,0	10,3	3582,2	10,3	3538,2	10,4	3584,7	10,3
78	3786,0	10,0	4006,6	9,7	4067,9	9,6	4014,5	9,7	4064,6	9,7
79	4303,2	9,3	4554,3	9,1	4620,8	9,0	4556,5	9,1	4610,2	9,1
80	4891,8	8,7	5177,6	8,5	5249,8	8,4	5172,5	8,5	5229,9	8,5
81	5560,8	8,1	5886,2	7,9	5964,3	7,9	5871,6	7,9	5932,8	7,9
82	6320,2	7,6	6690,4	7,3	6774,7	7,3	6663,9	7,4	6728,9	7,4
83	7180,6	7,0	7601,7	6,8	7692,4	6,8	7560,4	6,9	7629,0	6,8
84	8153,8	6,5	8632,5	6,3	8729,7	6,3	8572,9	6,4	8644,8	6,3
85	9252,3	6,0	9796,2	5,8	9899,9	5,8	9714,0	5,9	9789,0	5,9
86	10489,5	5,6	11106,8	5,4	11217,0	5,4	10997,3	5,4	11074,8	5,4
87	11879,5	5,1	12579,4	5,0	12695,9	4,9	12437,0	5,0	12516,1	5,0
88	13437,1	4,7	14229,8	4,6	14352,0	4,5	14047,8	4,6	14127,7	4,6
89	15177,6	4,3	16074,0	4,2	16201,4	4,2	15844,8	4,2	15924,3	4,2
90	17121,4	4,0	18133,8	3,8	18265,5	3,8	17848,8	3,9	17926,2	3,9
91	19309,1	3,6	20452,2	3,5	20587,1	3,5	20100,9	3,5	20174,6	3,5
92	21776,3	3,3	23067,0	3,2	23203,9	3,1	22637,2	3,2	22704,9	3,2
93	24558,8	3,0	26016,1	2,8	26153,2	2,8	25493,5	2,9	25552,5	2,9
94	27696,8	2,6	29342,3	2,5	29477,4	2,5	28710,2	2,6	28757,4	2,6
95	31235,8	2,3	33093,7	2,2	33224,1	2,2	32332,8	2,3	32364,1	2,3
96	35227,0	2,0	37324,7	1,9	37447,1	1,9	36412,4	1,9	36423,3	1,9
97	39728,1	1,6	42096,7	1,6	42206,8	1,6	41006,9	1,6	40991,5	1,6
98	44804,4	1,1	47478,8	1,1	47571,6	1,1	46181,0	1,1	46132,7	1,1
99	50529,3	0,5	53548,9	0,5	53618,2	0,5	52008,0	0,5	51918,7	0,5
