# Supplementary material for: Deprivation-specific life tables using multivariable flexible modelling – trends from 2000–2002 to 2010–2012, Portugal
Source: BMC Public Health. 2019 Mar 7;19:276. doi: 10.1186/s12889-019-6579-6 (PMC6407195; doi:10.1186/s12889-019-6579-6)
Supplement: Supplementary file 4 — Table S3. Life tables by deprivation quintile for men in the period 2010–2012. (PDF 411 kb) [file 12889_2019_6579_MOESM4_ESM.pdf]

Table S3 - Life tables by deprivation quintile (1-Least deprived) for men in the period 2010-2012 (m_x - mortality rate; e_x - life expectancy at age x).

age	EDI = 1		EDI = 2		EDI = 3		EDI = 4		EDI = 5	
	m_x	e_x	m_x	e_x	m_x	e_x	m_x	e_x	m_x	e_x
0	185,4	78,8	238,7	77,8	243,9	77,5	273,5	77,4	322,3	76,7
1	27,7	77,9	35,5	76,9	36,3	76,7	40,6	76,6	47,8	76,0
2	15,6	76,9	20,0	76,0	20,5	75,7	22,9	75,6	26,9	75,0
3	12,7	75,9	16,2	75,0	16,6	74,7	18,5	74,6	21,7	74,0
4	10,7	75,0	13,6	74,0	13,9	73,7	15,5	73,6	18,1	73,0
5	9,3	74,0	11,9	73,0	12,1	72,7	13,5	72,7	15,8	72,1
6	8,5	73,0	10,7	72,0	11,0	71,7	12,2	71,7	14,2	71,1
7	8,0	72,0	10,1	71,0	10,3	70,8	11,4	70,7	13,3	70,1
8	7,8	71,0	9,9	70,0	10,1	69,8	11,2	69,7	13,0	69,1
9	8,0	70,0	10,0	69,0	10,3	68,8	11,3	68,7	13,2	68,1
10	8,5	69,0	10,6	68,0	10,9	67,8	12,0	67,7	13,9	67,1
11	9,3	68,0	11,7	67,1	12,0	66,8	13,2	66,7	15,2	66,1
12	10,7	67,0	13,3	66,1	13,6	65,8	15,0	65,7	17,3	65,1
13	12,5	66,0	15,6	65,1	15,9	64,8	17,5	64,7	20,1	64,1
14	14,9	65,0	18,6	64,1	19,0	63,8	20,8	63,7	23,9	63,1
15	18,1	64,0	22,4	63,1	22,9	62,8	25,1	62,8	28,8	62,2
16	22,0	63,0	27,2	62,1	27,8	61,8	30,4	61,8	34,8	61,2
17	26,7	62,1	33,0	61,1	33,7	60,9	36,8	60,8	42,1	60,2
18	32,2	61,1	39,7	60,1	40,6	59,9	44,1	59,8	50,4	59,2
19	38,2	60,1	46,9	59,2	48,0	58,9	52,2	58,8	59,5	58,3
20	44,3	59,1	54,4	58,2	55,6	57,9	60,3	57,9	68,7	57,3
21	50,1	58,1	61,2	57,2	62,6	57,0	67,8	56,9	77,1	56,3
22	55,1	57,2	67,3	56,3	68,8	56,0	74,4	55,9	84,4	55,4
23	59,4	56,2	72,3	55,3	74,0	55,0	79,9	55,0	90,5	54,4
24	63,0	55,2	76,5	54,3	78,3	54,1	84,3	54,0	95,4	53,5
25	66,0	54,3	79,9	53,4	81,8	53,1	87,9	53,1	99,3	52,5
26	68,5	53,3	82,7	52,4	84,7	52,2	90,9	52,1	102,5	51,6
27	70,8	52,3	85,3	51,5	87,3	51,2	93,6	51,2	105,3	50,6
28	73,1	51,4	87,9	50,5	90,0	50,2	96,3	50,2	108,2	49,7
29	75,9	50,4	91,0	49,5	93,1	49,3	99,5	49,3	111,6	48,7
30	79,3	49,5	94,9	48,6	97,1	48,3	103,6	48,3	116,0	47,8
31	83,9	48,5	100,1	47,6	102,5	47,4	109,2	47,4	122,0	46,8
32	89,7	47,5	106,8	46,7	109,3	46,4	116,2	46,4	129,6	45,9
33	96,7	46,6	114,8	45,7	117,6	45,5	124,8	45,5	139,0	45,0
34	105,0	45,6	124,4	44,8	127,3	44,5	134,9	44,5	150,0	44,0
35	114,6	44,7	135,5	43,8	138,7	43,6	146,7	43,6	162,9	43,1
36	125,6	43,7	148,0	42,9	151,6	42,7	160,1	42,6	177,4	42,2
37	137,8	42,8	162,1	42,0	166,0	41,7	175,0	41,7	193,6	41,2
38	151,3	41,8	177,5	41,0	181,7	40,8	191,3	40,8	211,3	40,3
39	165,8	40,9	194,0	40,1	198,7	39,9	208,8	39,9	230,2	39,4
40	181,1	40,0	211,4	39,2	216,5	38,9	227,1	38,9	250,0	38,5
41	196,8	39,0	229,2	38,3	234,8	38,0	245,9	38,0	270,2	37,6
42	213,0	38,1	247,5	37,3	253,5	37,1	265,0	37,1	290,7	36,7
43	229,7	37,2	266,2	36,4	272,7	36,2	284,6	36,2	311,6	35,8
44	246,9	36,3	285,4	35,5	292,4	35,3	304,7	35,3	333,1	34,9
45	264,8	35,4	305,4	34,6	312,8	34,4	325,5	34,4	355,2	34,0
46	283,6	34,5	326,2	33,7	334,1	33,5	347,1	33,5	378,1	33,1
47	303,3	33,5	348,0	32,8	356,6	32,6	369,8	32,6	402,1	32,2
48	324,4	32,6	371,3	32,0	380,4	31,7	393,8	31,8	427,5	31,4
49	347,1	31,8	396,3	31,1	406,0	30,8	419,7	30,9	454,8	30,5

Table S3 (cont.) - Life tables by deprivation quintile (1-Least deprived) for men in the period 2010-2012 (m_x - mortality rate; e_x - life expectancy at age x).

age	EDI = 1		EDI = 2		EDI = 3		EDI = 4		EDI = 5	
	m_x	e_x	m_x	e_x	m_x	e_x	m_x	e_x	m_x	e_x
50	371,8	30,9	423,5	30,2	433,9	30,0	447,7	30,0	484,4	29,6
51	398,9	30,0	453,3	29,3	464,5	29,1	478,5	29,2	516,8	28,8
52	428,9	29,1	486,1	28,5	498,1	28,2	512,3	28,3	552,3	27,9
53	461,9	28,2	522,2	27,6	535,2	27,4	549,5	27,4	591,4	27,1
54	498,3	27,3	562,0	26,7	576,0	26,5	590,4	26,6	634,4	26,2
55	538,5	26,5	605,9	25,9	621,0	25,7	635,5	25,7	681,7	25,4
56	583,0	25,6	654,3	25,0	670,7	24,8	685,1	24,9	733,7	24,6
57	632,1	24,8	707,8	24,2	725,5	24,0	739,9	24,1	791,0	23,8
58	686,6	23,9	766,8	23,4	786,1	23,2	800,4	23,2	854,2	22,9
59	746,9	23,1	832,1	22,5	853,1	22,3	867,1	22,4	923,8	22,1
60	813,8	22,2	904,4	21,7	927,2	21,5	941,0	21,6	1000,8	21,3
61	888,0	21,4	984,5	20,9	1009,4	20,7	1022,6	20,8	1085,7	20,5
62	970,4	20,6	1073,2	20,1	1100,4	19,9	1113,0	20,0	1179,7	19,8
63	1062,0	19,8	1171,6	19,3	1201,4	19,1	1213,1	19,2	1283,6	19,0
64	1163,9	19,0	1280,9	18,6	1313,5	18,4	1324,1	18,5	1398,7	18,2
65	1277,3	18,2	1402,3	17,8	1438,1	17,6	1447,2	17,7	1526,1	17,5
66	1403,7	17,5	1537,2	17,0	1576,6	16,8	1584,0	17,0	1667,5	16,7
67	1544,6	16,7	1687,4	16,3	1730,7	16,1	1735,9	16,2	1824,3	16,0
68	1701,8	16,0	1854,6	15,6	1902,3	15,4	1904,9	15,5	1998,4	15,3
69	1877,4	15,2	2040,9	14,8	2093,5	14,7	2092,8	14,8	2191,9	14,6
70	2073,6	14,5	2248,6	14,1	2306,7	14,0	2302,1	14,1	2407,0	13,9
71	2292,9	13,8	2480,4	13,4	2544,6	13,3	2535,3	13,4	2646,3	13,2
72	2538,4	13,1	2739,1	12,8	2810,2	12,6	2795,3	12,7	2912,7	12,6
73	2813,2	12,4	3028,2	12,1	3107,0	12,0	3085,4	12,1	3209,4	11,9
74	3121,1	11,8	3351,4	11,5	3438,7	11,3	3409,2	11,4	3540,2	11,3
75	3466,3	11,1	3712,9	10,8	3809,9	10,7	3770,9	10,8	3909,1	10,7
76	3853,5	10,5	4117,6	10,2	4225,4	10,1	4175,2	10,2	4320,8	10,1
77	4288,1	9,9	4570,7	9,6	4690,6	9,5	4627,2	9,6	4780,4	9,5
78	4776,2	9,3	5078,5	9,1	5212,0	8,9	5132,9	9,1	5293,8	9,0
79	5324,5	8,7	5647,6	8,5	5796,4	8,4	5699,1	8,5	5867,6	8,4
80	5941,0	8,2	6286,0	8,0	6452,0	7,9	6333,1	8,0	6509,2	7,9
81	6634,2	7,6	7002,3	7,5	7187,6	7,3	7043,4	7,5	7227,0	7,4
82	7414,3	7,1	7806,4	7,0	8013,4	6,9	7839,6	7,0	8030,2	6,9
83	8292,3	6,6	8709,5	6,5	8940,9	6,4	8732,4	6,5	8929,4	6,5
84	9281,0	6,2	9724,0	6,0	9982,9	5,9	9733,9	6,1	9936,5	6,0
85	10394,7	5,7	10864,1	5,6	11154,0	5,5	10857,8	5,6	11064,8	5,6
86	11649,6	5,3	12145,8	5,2	12470,7	5,1	12119,2	5,2	12329,2	5,2
87	13064,1	4,9	13587,1	4,8	13951,3	4,7	13535,6	4,8	13746,7	4,8
88	14658,9	4,5	15208,3	4,4	15616,9	4,3	15126,4	4,5	15336,0	4,4
89	16456,0	4,1	17030,9	4,1	17489,4	4,0	16911,9	4,1	17116,9	4,1
90	18475,1	3,8	19073,5	3,7	19588,1	3,7	18909,8	3,8	19106,4	3,7
91	20741,9	3,5	21361,2	3,4	21938,8	3,4	21143,9	3,4	21327,2	3,4
92	23286,8	3,2	23923,3	3,1	24571,5	3,1	23641,8	3,1	23806,1	3,1
93	26144,0	2,8	26792,6	2,8	27520,1	2,8	26434,9	2,8	26573,1	2,8
94	29351,8	2,6	30006,1	2,5	30822,6	2,5	29557,9	2,5	29661,7	2,5
95	32953,1	2,2	33605,1	2,2	34521,4	2,2	33049,9	2,3	33109,3	2,3
96	36996,3	1,9	37635,6	1,9	38664,0	1,9	36954,4	1,9	36957,6	1,9
97	41535,6	1,6	42149,7	1,6	43303,8	1,5	41320,3	1,6	41253,2	1,6
98	46631,9	1,1	47205,1	1,1	48500,4	1,1	46201,9	1,1	46048,1	1,1
99	52353,4	0,5	52866,8	0,5	54320,6	0,5	51660,2	0,5	51400,4	0,5
