# Supplementary material for: Deprivation-specific life tables using multivariable flexible modelling – trends from 2000–2002 to 2010–2012, Portugal
Source: BMC Public Health. 2019 Mar 7;19:276. doi: 10.1186/s12889-019-6579-6 (PMC6407195; doi:10.1186/s12889-019-6579-6)
Supplement: Supplementary file 5 — Table S4. Life tables by deprivation quintile for women in the period 2010–2012. (PDF 411 kb) [file 12889_2019_6579_MOESM5_ESM.pdf]

Table S4 - Life tables by deprivation quintile (1-Least deprived) for women in the period 2010-2012 (m_x - mortality rate; e_x - life expectancy at age x).

age	EDI = 1		EDI = 2		EDI = 3		EDI = 4		EDI = 5	
	m_x	e_x	m_x	e_x	m_x	e_x	m_x	e_x	m_x	e_x
0	263,6	84,3	278,3	83,8	301,2	83,6	316,3	83,6	339,4	83,4
1	28,5	83,6	30,1	83,1	32,5	82,8	34,1	82,9	36,6	82,7
2	18,0	82,6	19,0	82,1	20,5	81,8	21,5	81,9	23,1	81,7
3	17,1	81,6	18,1	81,1	19,5	80,8	20,4	80,9	21,9	80,7
4	15,7	80,6	16,6	80,1	17,9	79,9	18,8	79,9	20,1	79,8
5	14,1	79,6	14,9	79,1	16,1	78,9	16,8	79,0	18,0	78,8
6	12,6	78,6	13,3	78,2	14,3	77,9	15,0	78,0	16,0	77,8
7	11,2	77,7	11,8	77,2	12,8	76,9	13,3	77,0	14,2	76,8
8	10,1	76,7	10,7	76,2	11,5	75,9	12,0	76,0	12,8	75,8
9	9,3	75,7	9,8	75,2	10,6	74,9	11,0	75,0	11,8	74,8
10	8,9	74,7	9,4	74,2	10,1	73,9	10,5	74,0	11,2	73,8
11	8,9	73,7	9,4	73,2	10,1	72,9	10,5	73,0	11,2	72,8
12	9,2	72,7	9,7	72,2	10,4	71,9	10,9	72,0	11,6	71,8
13	9,8	71,7	10,4	71,2	11,2	70,9	11,6	71,0	12,3	70,8
14	10,8	70,7	11,4	70,2	12,2	70,0	12,7	70,0	13,5	69,9
15	12,0	69,7	12,7	69,2	13,6	69,0	14,1	69,1	15,0	68,9
16	13,6	68,7	14,3	68,2	15,4	68,0	15,9	68,1	16,9	67,9
17	15,3	67,7	16,2	67,2	17,3	67,0	18,0	67,1	19,0	66,9
18	17,2	66,7	18,2	66,3	19,5	66,0	20,2	66,1	21,4	65,9
19	19,2	65,8	20,3	65,3	21,7	65,0	22,4	65,1	23,8	64,9
20	21,0	64,8	22,2	64,3	23,7	64,0	24,5	64,1	25,9	63,9
21	22,4	63,8	23,7	63,3	25,3	63,0	26,1	63,1	27,6	62,9
22	23,4	62,8	24,7	62,3	26,4	62,1	27,2	62,1	28,8	62,0
23	24,1	61,8	25,4	61,3	27,1	61,1	27,9	61,2	29,5	61,0
24	24,5	60,8	25,9	60,3	27,6	60,1	28,4	60,2	30,0	60,0
25	24,7	59,8	26,1	59,4	27,8	59,1	28,6	59,2	30,2	59,0
26	24,9	58,9	26,4	58,4	28,1	58,1	28,8	58,2	30,4	58,0
27	25,2	57,9	26,7	57,4	28,4	57,1	29,1	57,2	30,7	57,1
28	25,7	56,9	27,2	56,4	28,9	56,2	29,7	56,2	31,3	56,1
29	26,6	55,9	28,1	55,4	29,9	55,2	30,6	55,3	32,2	55,1
30	28,0	54,9	29,6	54,4	31,4	54,2	32,1	54,3	33,8	54,1
31	30,0	53,9	31,7	53,4	33,7	53,2	34,4	53,3	36,2	53,1
32	32,8	52,9	34,7	52,5	36,8	52,2	37,6	52,3	39,5	52,1
33	36,4	52,0	38,5	51,5	40,8	51,2	41,7	51,3	43,8	51,2
34	40,9	51,0	43,2	50,5	45,8	50,3	46,7	50,4	49,0	50,2
35	46,2	50,0	48,9	49,5	51,7	49,3	52,8	49,4	55,3	49,2
36	52,5	49,0	55,5	48,6	58,7	48,3	59,8	48,4	62,7	48,2
37	59,6	48,0	63,1	47,6	66,6	47,3	67,8	47,4	71,0	47,3
38	67,4	47,1	71,4	46,6	75,3	46,4	76,7	46,5	80,2	46,3
39	75,8	46,1	80,2	45,6	84,6	45,4	86,0	45,5	89,9	45,3
40	84,2	45,1	89,2	44,7	94,0	44,4	95,5	44,5	99,8	44,4
41	92,4	44,2	97,8	43,7	103,0	43,5	104,6	43,6	109,2	43,4
42	100,1	43,2	105,9	42,8	111,5	42,5	113,1	42,6	118,0	42,5
43	107,2	42,3	113,5	41,8	119,4	41,6	121,0	41,7	126,2	41,5
44	114,0	41,3	120,6	40,8	126,9	40,6	128,4	40,7	133,8	40,6
45	120,3	40,4	127,4	39,9	133,8	39,7	135,4	39,8	141,0	39,6
46	126,4	39,4	133,9	38,9	140,6	38,7	142,1	38,8	147,9	38,7
47	132,5	38,4	140,3	38,0	147,2	37,8	148,7	37,9	154,7	37,7
48	138,8	37,5	147,0	37,1	154,1	36,8	155,6	36,9	161,7	36,8
49	145,6	36,6	154,2	36,1	161,6	35,9	162,9	36,0	169,2	35,9

Table S4 (cont.) - Life tables by deprivation quintile (1-Least deprived) for women in the period 2010-2012 (m_x - mortality rate; e_x - life expectancy at age x).

age	EDI = 1		EDI = 2		EDI = 3		EDI = 4		EDI = 5	
	m_x	e_x	m_x	e_x	m_x	e_x	m_x	e_x	m_x	e_x
50	153,2	35,6	162,2	35,2	169,9	34,9	171,2	35,1	177,7	34,9
51	162,0	34,7	171,6	34,2	179,6	34,0	180,8	34,1	187,5	34,0
52	172,2	33,7	182,4	33,3	190,8	33,1	191,9	33,2	198,9	33,0
53	184,0	32,8	194,9	32,3	203,7	32,1	204,7	32,2	212,0	32,1
54	197,5	31,8	209,2	31,4	218,5	31,2	219,4	31,3	227,1	31,2
55	212,9	30,9	225,6	30,5	235,5	30,3	236,3	30,4	244,4	30,2
56	230,6	30,0	244,3	29,5	254,9	29,3	255,5	29,4	264,1	29,3
57	250,8	29,0	265,7	28,6	277,0	28,4	277,5	28,5	286,7	28,4
58	273,9	28,1	290,2	27,7	302,3	27,5	302,6	27,6	312,4	27,5
59	300,2	27,2	318,1	26,8	331,3	26,6	331,3	26,7	341,7	26,5
60	330,3	26,3	350,1	25,8	364,3	25,6	364,0	25,8	375,2	25,6
61	364,8	25,3	386,6	24,9	402,0	24,7	401,4	24,9	413,5	24,7
62	404,1	24,4	428,4	24,0	445,1	23,8	444,1	24,0	457,2	23,8
63	449,2	23,5	476,1	23,1	494,4	22,9	492,9	23,1	507,1	22,9
64	500,8	22,6	530,8	22,2	550,9	22,1	548,7	22,2	564,1	22,1
65	559,8	21,7	593,5	21,3	615,5	21,2	612,5	21,3	629,3	21,2
66	627,5	20,9	665,3	20,5	689,5	20,3	685,6	20,4	703,9	20,3
67	705,1	20,0	747,6	19,6	774,3	19,4	769,3	19,6	789,3	19,4
68	794,1	19,1	842,1	18,7	871,5	18,6	865,2	18,7	887,1	18,6
69	896,3	18,3	950,5	17,9	983,1	17,7	975,1	17,9	999,1	17,8
70	1013,6	17,4	1074,9	17,1	1111,0	16,9	1101,1	17,0	1127,5	16,9
71	1148,2	16,6	1217,7	16,2	1257,9	16,1	1245,7	16,2	1274,6	16,1
72	1302,8	15,8	1381,8	15,4	1426,4	15,3	1411,4	15,4	1443,2	15,3
73	1480,3	15,0	1570,2	14,6	1619,8	14,5	1601,4	14,6	1636,5	14,5
74	1684,1	14,2	1786,4	13,9	1841,7	13,7	1819,3	13,8	1857,8	13,8
75	1918,0	13,4	2034,6	13,1	2096,2	13,0	2069,0	13,1	2111,4	13,0
76	2186,2	12,7	2319,4	12,4	2388,0	12,2	2355,0	12,4	2401,7	12,3
77	2493,8	12,0	2645,9	11,6	2722,3	11,5	2682,5	11,6	2733,9	11,6
78	2846,2	11,3	3019,9	10,9	3105,1	10,8	3057,2	10,9	3113,6	10,9
79	3249,5	10,6	3448,1	10,3	3543,0	10,2	3485,4	10,3	3547,4	10,2
80	3710,6	9,9	3937,6	9,6	4043,3	9,5	3974,4	9,6	4042,4	9,6
81	4237,2	9,2	4496,7	9,0	4614,4	8,9	4532,0	9,0	4606,4	8,9
82	4837,7	8,6	5134,3	8,4	5265,2	8,3	5166,9	8,4	5248,2	8,3
83	5521,4	8,0	5860,3	7,8	6005,7	7,7	5888,8	7,8	5977,4	7,7
84	6298,4	7,5	6685,5	7,2	6846,8	7,1	6708,0	7,2	6804,4	7,2
85	7179,7	6,9	7621,5	6,7	7800,3	6,6	7635,8	6,7	7740,3	6,7
86	8177,2	6,4	8680,9	6,2	8878,7	6,1	8684,4	6,2	8797,3	6,2
87	9303,5	5,9	9877,3	5,7	10095,6	5,6	9866,5	5,7	9988,1	5,7
88	10572,0	5,4	11224,7	5,2	11465,3	5,2	11195,9	5,3	11326,2	5,2
89	11996,5	5,0	12738,1	4,8	13002,5	4,7	12686,5	4,8	12825,6	4,8
90	13595,6	4,5	14436,9	4,4	14726,8	4,3	14357,1	4,4	14504,8	4,4
91	15403,7	4,1	16358,0	4,0	16675,5	3,9	16243,4	4,0	16399,5	4,0
92	17452,2	3,7	18534,7	3,6	18882,0	3,6	18377,6	3,6	18541,7	3,6
93	19773,2	3,3	21001,0	3,2	21380,4	3,2	20792,2	3,2	20963,7	3,2
94	22402,9	3,0	23795,6	2,9	24209,4	2,8	23524,0	2,9	23702,1	2,9
95	25382,3	2,6	26962,0	2,5	27412,8	2,5	26614,7	2,5	26798,2	2,5
96	28758,0	2,2	30549,7	2,1	31040,0	2,1	30111,5	2,1	30298,8	2,1
97	32582,6	1,7	34614,9	1,7	35147,2	1,7	34067,7	1,7	34256,6	1,7
98	36915,8	1,2	39220,9	1,2	39797,8	1,2	38543,8	1,2	38731,4	1,2
99	41825,3	0,5	44439,9	0,5	45063,8	0,5	43607,9	0,5	43790,8	0,5
